# IFD-YOLO: A Lightweight Infrared Sensor-Based Detector for Small UAV Targets

**DOI:** 10.3390/s25247449

**Published:** 2025-12-07

**Authors:** Fu Li, Xuehan Lv, Ming Zhao, Wangyu Wu

**Affiliations:** 1 School of Internet of Things Engineering, Wuxi University, Wuxi 214105, China; lifu@cwxu.edu.cn; 2 School of Computer Science and Technology, Nanjing University of Information Science and Technology, Nanjing 210044, China; lvxh18263415990@163.com; 3 School of Cyber Science and Engineering, Wuxi University, Wuxi 214105, China; 4 School of Computer Science, University of Liverpool, Liverpool L69 3DR, UK; wangyu.wu@liverpool.ac.uk

**Keywords:** UAV, infrared, small target, DyGhost, YOLOv11

## Abstract

**Highlights:**

**What are the main findings?**

- A novel lightweight infrared small-target detection model, IFD-YOLO, is developed based on YOLOv11n. It integrates the RepViT backbone with two newly proposed components—the C3k2-DyGhost module and the Adaptive Fusion-IoU (AF-IoU) loss—to enhance feature extraction, dynamic adaptability, and bounding-box regression accuracy.
- Extensive experiments on benchmark infrared datasets confirm that IFD-YOLO achieves higher detection accuracy and better efficiency than existing lightweight detectors, demonstrating strong robustness under complex UAV infrared scenarios.

**What are the implications of the main findings?**

- The proposed architecture provides a well-balanced solution between detection accuracy, computational cost, and model compactness, making it well-suited for lightweight and real-time infrared perception tasks.
- The design concept offers valuable insights for future embedded adaptation and hardware–software co-optimization of lightweight infrared detection algorithms in UAV-related applications.

**Abstract:**

The detection of small targets in infrared imagery captured by unmanned aerial vehicles (UAVs) is critical for surveillance and monitoring applications. However, this task is challenged by the small target size, low signal-to-noise ratio, and the limited computational resources of UAV platforms. To address these issues, this paper proposes IFD-YOLO, a novel lightweight detector based on YOLOv11n, specifically designed for onboard infrared sensing systems. Our framework introduces three key improvements. First, a RepViT backbone enhances both global and local feature extraction. Second, a C3k2-DyGhost module performs dynamic and efficient feature fusion. Third, an Adaptive Fusion-IoU (AF-IoU) loss improves bounding-box regression accuracy for small targets. Extensive experiments on the HIT-UAV and IRSTD-1k datasets demonstrate that IFD-YOLO achieves a superior balance between accuracy and efficiency. Compared to YOLOv11n, our model improves mAP@50 and mAP@50:95 by 4.9% and 3.1%, respectively, while simultaneously reducing the number of parameters and GFLOPs by 23% and 21%. These results validate the strong potential of IFD-YOLO for real-time infrared sensing tasks on resource-constrained UAV platforms.

## 1. Introduction

With the rapid development of unmanned aerial vehicle (UAV) technology, UAVs have been widely applied in various fields such as national defense reconnaissance, maritime surveillance, disaster rescue, and border patrol. Owing to their advantages of high mobility, flexible deployment, and low operational cost, UAVs are capable of efficiently performing missions in complex or hazardous environments, greatly enhancing aerial monitoring and information acquisition capabilities.

Among various sensing modalities, infrared imaging (IR) [1] has become a crucial technology for UAV-based monitoring and recognition tasks due to its strong resistance to illumination interference, all-day operability, and high environmental adaptability. Infrared imaging detects targets based on thermal radiation, allowing it to function reliably even in low-visibility conditions such as nighttime, fog, or smoke. This capability makes it highly suitable for tasks such as military reconnaissance, search and rescue, and surveillance in challenging environments.

However, UAV-based infrared imagery introduces several unique challenges. Due to the high flight altitude and long imaging distance, targets usually appear as small and indistinct objects in the imaging plane. In addition, the inherent characteristics of infrared imaging—such as low resolution, weak thermal signatures, and cluttered backgrounds—lead to low contrast and blurred target boundaries, making small objects easily confused with background noise and resulting in a low signal-to-noise ratio. These factors significantly affect detection accuracy and stability.

At the same time, UAV onboard devices face strict limitations in computational capacity and power consumption. These constraints require detection algorithms to be lightweight and capable of real-time inference while still maintaining reliable performance in complex infrared environments. Against this backdrop, one-stage detectors have become particularly suitable for infrared small target detection due to their compact architecture and high computational efficiency. Among them, YOLO (You Only Look Once) series has emerged as the mainstream solution owing to its end-to-end prediction mechanism and remarkable inference speed. Compared with other one-stage detectors such as SSD (Single Shot MultiBox Detector) [2] and RetinaNet [3], YOLO demonstrates superior performance in inference speed, model compactness, and deployment efficiency. With the continuous evolution of the YOLO family (e.g., YOLOv5, YOLOv8, YOLOv11), its feature extraction and small-target perception capabilities in infrared scenarios have been significantly enhanced. Overall, YOLO series provides an efficient solution that achieves a favorable balance between detection speed and accuracy for UAV-based infrared small target detection.

The YOLOv11n [4] model achieves a favorable balance between detection accuracy and computational efficiency by optimizing its feature extraction modules and integrating attention mechanisms. With its lightweight and efficient design, YOLOv11n is well-suited for real-time object detection tasks. Building upon this foundation, this paper proposes an improved infrared small target detection model named IFD-YOLO, which effectively enhances detection accuracy while reducing both the number of parameters and computational cost. The main contributions of this study are summarized as follows:

(a) RepViT Backbone Reconstruction: We introduce the RepViT [5] backbone into the YOLOv11 framework. Leveraging the collaborative effects of the MetaFormer [6] architecture and structural re-parameterization mechanism, this backbone achieves efficient fusion of local spatial information and global contextual relationships during the feature extraction stage. This design substantially enhances feature perception and discrimination capability, thereby improving detection performance for infrared small targets.
(b) Dynamic Ghost Feature Extraction Module (C3k2_DyGhost): Building on the original C3k2 structure, we integrate the Ghost [7] feature generation mechanism with dynamic convolution to form a new feature extraction module, termed C3k2_DyGhost. This module adaptively generates additional feature maps and adjusts convolutional kernel weights according to the input feature distribution. As a result, the module enhances the representational quality of the extracted features and reduces degradation caused by low-contrast and cluttered infrared backgrounds. Consequently, it enhances both detection accuracy and generalization capability for small infrared targets.
(c) Adaptive Fusion-IoU Loss (AF-IoU): We propose an Adaptive Fusion-IoU loss function to replace the conventional bounding box regression loss. This loss dynamically adjusts the weighting between high- and low-quality predicted boxes through an adaptive weighting mechanism, enabling a smooth balance during the training process. In infrared small-target detection, AF-IoU improves the stability and accuracy of bounding-box regression for small, low-contrast targets, leading to better overall detection performance.

## 2. Related Work

Before the emergence of deep learning methods, infrared target detection mainly relied on traditional image processing and pattern recognition techniques. These approaches typically detect targets by using differences in grayscale, texture, or spatial distribution between the target and the background, and they rely on handcrafted features and manually designed decision rules. Common methods included the use of spatial or frequency domain filters to suppress background noise and enhance target responses, such as high-pass filtering [8], Laplacian operators [9], Top-hat filtering [10], and Laplacian of Gaussian (LoG) filtering [11]. Although these methods feature simple structures and high computational efficiency, they are prone to false detections in heavily cluttered or noisy scenes. For example, Zhao et al. [12,13] proposed contrast-based and gradient-fusion anomaly-detection methods that can suppress background interference and enhance weak infrared targets. However, their performance degrades in dynamic UAV infrared scenes.

Another class of methods is based on statistical modeling principles, which achieve target-background separation by constructing statistical models of the background distribution or by detecting pixel-level intensity anomalies. Representative techniques include background-prediction–based anomaly detection [14] (e.g., RX), local-statistics–based measures, and Bayesian modeling [15]. These methods perform well under static or stationary background conditions but struggle to handle dynamic scenes or cluttered environments. In addition, local contrast-based methods have been widely adopted. Their core idea is to exploit the differences in brightness or energy between the target region and its surrounding background to achieve detection. Typical examples include the Local Contrast Measure (LCM) and Local Energy Contrast (LEC). However, because these methods depend on fixed handcrafted features and thresholds, their performance degrades notably with changes in imaging conditions, target scale, or background complexity. Consequently, their robustness deteriorates significantly in multi-scene or low signal-to-noise ratio (SNR) environments. These limitations have motivated the development of deep-learning-based infrared detection methods and provided guidance for designing more robust algorithms.

With the rapid advancement of deep learning, object detection technology has become increasingly mature. Deep learning has substantially improved the handling of several long-standing challenges in infrared small-target detection. In parallel, recent studies on hyperspectral video tracking networks has provided valuable architectural insights for infrared small-target detection. Recently, Zhao et al. [16] proposed SASU-Net, a spectral adaptive aggregation and scale-updating network that improves feature stability and tracking performance under illumination variations, offering valuable guidance for lightweight infrared feature fusion in detection tasks. Another representative framework is OCSCNet-Tracker [17], which integrates octave convolution with a spatial–spectral capsule structure to capture hierarchical context and enhance spectral–spatial representation. Furthermore, SRTE-Net [18] introduces a spectral–spatial similarity-reduction and reorganized-texture-encoding strategy, which effectively suppresses redundant spectral correlations while enhancing discriminative spatial texture representation in hyperspectral video tracking. Likewise, Jiang et al. [19] proposed a cross-modal spectral complementary and memory prompt network to further strengthen spectral–spatial interaction and tracking stability in hyperspectral videos.

Currently, mainstream object detection algorithms can be broadly categorized into two-stage and one-stage approaches. In the context of UAV-based infrared small target detection, two-stage methods—represented by the R-CNN [20] family (e.g., R-CNN and Fast R-CNN [21])—typically follow a two-step process. First, candidate regions are generated using heuristic algorithms such as Selective Search [22] or convolutional neural networks (CNNs) [23]. Then, object classification and bounding box regression are performed for each candidate region. Owing to their refined region proposal and analysis, two-stage methods achieve high detection accuracy. However, since this method relies on two stages of candidate region generation and precise detection, making them unsuitable for real-time detection in infrared scenarios.

In contrast, one-stage methods, represented by YOLO (You Only Look Once) [24] series, perform dense predictions directly over the entire image. By extracting features through CNNs and simultaneously performing object classification and bounding box regression, they achieve true end-to-end detection. This approach produces detection results in a single forward pass, significantly improving inference efficiency and reducing model complexity. Although slightly inferior in precision compared to two-stage methods, the lightweight structure and high efficiency of one-stage frameworks make them more advantageous for UAV-based infrared small target detection tasks.

However, in UAV infrared small target detection tasks, the inherent characteristics of infrared imaging, such as small target size, low contrast, and blurred edges, result from the nature of thermal radiation. In addition, complex thermal noise, false targets, and environmental factors such as climate, temperature, and sensor performance further complicate the imaging process. Consequently, existing methods still struggle to maintain high recognition accuracy and precision in such challenging scenarios.

To address these issues, numerous studies have been conducted in the field of infrared target detection for UAV aerial imagery. Yuan et al. [25] proposed the IRSDD-YOLOv5 model, which enhances detection accuracy by introducing an Infrared Small Target Detection Module (IRSTDM), optimizing the loss function, and constructing the SIDD dataset. However, this approach shows limited adaptability to dense multi-target scenarios, and its real-time performance decreases slightly. Wang et al. [26] developed the PHSI-RTDETR algorithm, which incorporates lightweight modules, HiLo attention, and the Inner-GIoU loss to achieve efficient detection. Nonetheless, its ability to extract semantic features of small targets remains insufficient in heavily occluded multi-class scenes. Aibibu et al. [27] proposed ERGW-Net, which improves detection performance through enhanced backbone and neck structures as well as a novel LGWPIoU loss, but its adaptability to weak-feature small targets in small-sample datasets remains limited, and extreme imaging conditions were not fully considered. Pan et al. [28] presented AIMED-Net, which enhances YOLOv7 by strengthening shallow and deep feature representations and introducing the sa-CIoU loss to improve infrared small target detection accuracy; however, its adaptability in densely occluded multi-target scenarios remains inadequate. Yan et al. [29] proposed DMF-YOLO, which integrates DDSConv, MFAM modules, and EW-BBRLF loss to optimize multi-scale detection. Although it performs well on public datasets, the increased number of parameters affects real-time performance on small UAVs. Liu et al. [30] introduced a Z-shaped cropping enhancement and improved YOLOv11 to boost confidence in occluded small target detection; however, its effectiveness in modeling irregular real-world occlusions and distinguishing complex scene categories still requires improvement. Moreover, Zhao et al. [31] proposed a spectral difference matching reduction framework that enhances deep feature perception for weak and small targets, providing valuable insights for designing lightweight detection models in infrared scenarios.

Overall, recent infrared small-target detection methods have achieved notable progress. However, under the constraints of UAV platforms they still fail to achieve a good trade-off among detection accuracy, model complexity, and real-time performance. To address these limitations, we propose IFD-YOLO, which is designed to provide lightweight feature extraction and more accurate bounding-box regression for infrared small targets.

## 3. The Proposed Method

### 3.1. Architecture

**Figure 1:**
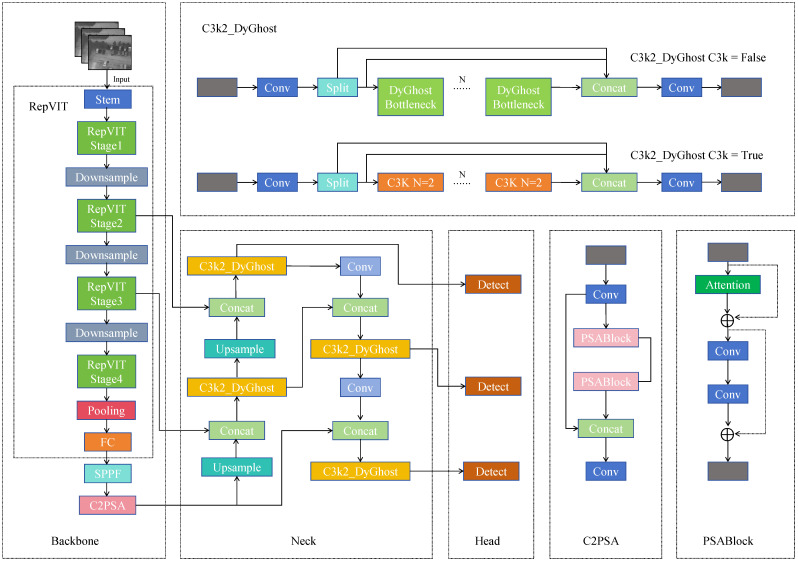
The IFD-YOLO network structure diagram. The model is based on YOLOv11, where the original backbone is replaced with RepViT for lightweight feature extraction, and the C3 modules in the neck are improved to C3k2_DyGhost for efficient feature fusion. In addition, the proposed AF-IoU loss is introduced during training to improve localization accuracy and convergence stability. Other components remain consistent with the YOLOv11 architecture.

This study aims to enhance the performance of YOLOv11n in UAV-based infrared small target detection tasks through targeted architectural improvements. The proposed model, named IFD-YOLO, specifically addresses three major challenges in infrared scenarios: blurred small-target features, strong background interference, and the trade-off between detection accuracy and inference speed. Overall, IFD-YOLO follows the typical one-stage detection architecture of YOLOv11, consisting of a backbone for feature extraction, a neck for multi-scale feature fusion, and a detection head for final prediction. As illustrated in Figure 1, IFD-YOLO improves three key components of YOLOv11n: the feature extractor, the feature enhancement module, and the bounding-box regression loss, which are redesigned as follows.

In the feature extraction stage, the original backbone network is replaced with the lightweight convolutional architecture RepViT (version M0.9). By decoupling the token mixer and channel mixer within a MetaFormer-like framework and introducing structural re-parameterization, RepViT strengthens feature extraction for infrared small targets. It helps the network capture subtle target cues more effectively than conventional backbones under low-contrast infrared conditions.

To further enhance feature representation capability, the original C3k2 module is replaced with the C3k2_DyGhost module. This module integrates dynamic convolution into the Ghost structure, allowing convolutional kernel weights to adapt dynamically according to the input feature distribution. Consequently, it improves fine-grained feature representation of infrared small targets while maintaining a lightweight architecture, and it enhances discrimination in complex backgrounds.

In the bounding box regression stage, an Adaptive Fusion-IoU (AF-IoU) loss function is employed to replace conventional IoU-based losses. AF-IoU introduces a Focal Box [32]–based dynamic scaling mechanism that adaptively adjusts the weights of prediction boxes with different quality levels. In addition, it employs an annealing strategy controlled by a ratio hyperparameter: the loss focuses more on low-quality boxes in the early training stages to accelerate convergence and gradually shifts its emphasis to high-quality boxes to improve regression precision. In addition, an optimized attention allocation mechanism is designed for infrared detection scenarios to alleviate the insensitivity of traditional IoU losses to small-target regression at high IoU thresholds, thereby improving localization accuracy.

In summary, IFD-YOLO achieves a well-balanced trade-off between detection accuracy and efficiency through the synergistic optimization of the backbone, feature transformation, and loss function. This framework provides a more effective solution for small target detection in complex infrared UAV scenarios.

### 3.2. RepViT Backbone Network

To enhance feature extraction capability and inference efficiency in UAV infrared small-target detection tasks, this study introduces the lightweight backbone network RepViT (Re-parameterized Vision Transformer) to replace the original convolutional backbone in YOLOv11. Infrared images are often characterized by small target sizes, low signal-to-noise ratios, and complex backgrounds. Traditional convolutional backbones, constrained by limited receptive fields, struggle to preserve fine-grained details and global semantic information during downsampling. Moreover, their large parameter counts and computational costs hinder real-time deployment on resource-constrained UAV platforms.

RepViT integrates the local feature extraction advantages of convolutional neural networks (CNNs) with the global modeling capability of Vision Transformers (ViTs) [33]. During training, it uses structural re-parameterization [34] to adopt a multi-branch architecture that enriches feature representation. At inference, these branches are fused into a single convolutional path, which reduces latency and computational cost while preserving detection accuracy. Similarly to the spectral–spatial interaction mechanism explored in SiamBSI [35], this design strengthens the joint modeling of local details and global semantics, enabling the network to capture fine-grained variations in small infrared targets under cluttered scenes.

**Figure 2:**
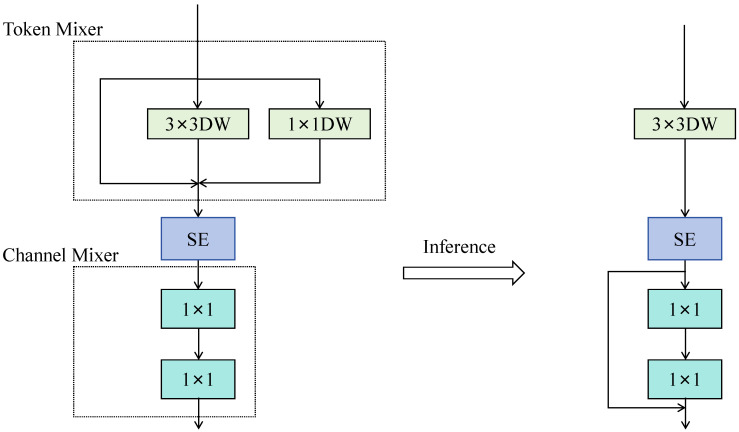
RepViTBlock structure.

**Figure 3:**
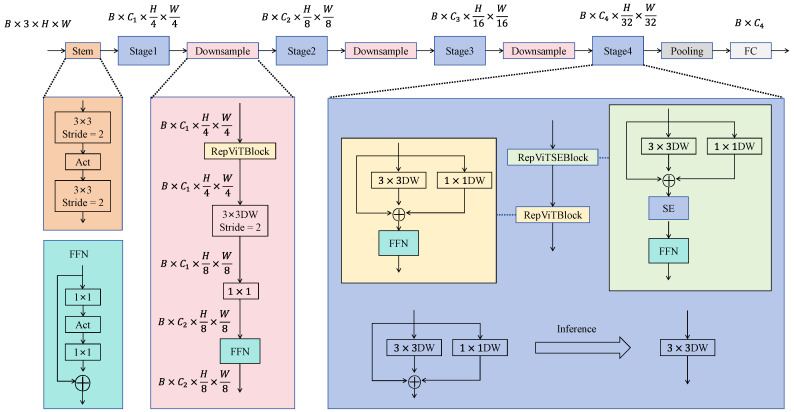
RepViT backbone architecture. The figure illustrates the hierarchical feature extraction process, including the Stem module, four sequential Stages, Downsample operations between stages, and the final pooling and fully connected layers. The detailed structures of the RepViTBlock and RepViTSEBlock are also illustrated, demonstrating the incorporation of depthwise convolutions, pointwise convolutions, and feed-forward network (FFN) components within the backbone.

The overall architecture of RepViT follows a hierarchical feature extraction strategy, consisting of three main components: the Stem, Stage, and Downsample modules, enabling progressive modeling from shallow local textures to deep semantic representations. In the Stem stage, the model first applies two 3 × 3 convolutions and a stride-2 downsampling operation to reduce spatial resolution and extract basic texture information, laying the foundation for subsequent high-level feature learning. Then, the features pass through multiple Stages and Downsample modules in an alternating manner for feature extraction and compression. Each Stage is composed of several RepViT Blocks or RepViT SE Blocks, as illustrated in Figure 2 and Figure 3, which show the structure of the RepViT Block and the overall RepViT network, respectively.

In each RepViT Block, the network performs feature modeling by combining depthwise convolution and pointwise convolution operations. Specifically, in the *i*-th RepViT Block of the *l*-th layer, the input feature is denoted as $X^{\left(l-1\right)}\in R^{H\times W\times C}$. The Token Mixer module performs spatial feature mixing and recombination through a 3 × 3 depthwise separable convolution, and its output can be formulated as:

(1) $$X_{token}^{\left(l\right)}=\sigma \left(BN\left({DWConv}_{3\times 3}\left(X^{\left(l-1\right)}\right)\right)\right)$$

where *DWConv*_3×3_ denotes the 3 × 3 depthwise convolution operation, *BN* represents batch normalization, and *σ*(·) is the ReLU activation function. Here, *DWConv*_3×3_ denotes a standard 3 × 3 depthwise convolution used in the DyGhost module, which is independent of the C3 or C3k2 block in YOLOv11. In addition, the Squeeze-and-Excitation (SE) [36] attention mechanism is incorporated to adaptively adjust channel weights, thereby emphasizing salient regional features of small targets in infrared images.

The Channel Mixer module consists of two 1 × 1 convolutional layers with GELU [37] activation functions. Functionally, it is equivalent to the Feed-Forward Network (FFN) [38] in Transformer architectures. By performing a process of “channel expansion–nonlinear mapping–channel compression,” it enables cross-channel feature modeling and nonlinear enhancement, thereby improving the richness and discriminative power of feature representations. Its computation can be expressed as:

(2) $$X_{ch}^{\left(l\right)}=W_{2}\cdot \delta \left(W_{1}\cdot X_{token}^{\left(l\right)}\right)$$

where $W_{1}$ and $W_{2}$ denote the weight matrices of the two 1 × 1 convolutional layers, and *δ*(·) represents the GELU activation function.

Finally, for blocks with *stride* = 1, a residual connection is introduced outside the Channel Mixer to preserve shallow feature integrity and alleviate the vanishing gradient problem. The output after incorporating the residual connection can be expressed as:

(3) $$X^{\left(l\right)}=X^{\left(l-1\right)}+X_{ch}^{\left(l\right)}$$

When stride = 1, the residual connection ensures that shallow texture information is preserved, mitigates gradient vanishing, and improves feature propagation efficiency.

Through this hierarchical modeling and multi-scale feature fusion strategy, RepViT effectively retains fine-grained features of infrared small targets during downsampling while capturing global contextual information. Consequently, it significantly enhances detection accuracy and feature adaptability under a lightweight design.

Finally, the backbone network outputs multi-scale feature maps to provide complementary high-resolution and high-semantic features for the YOLO detection head, thereby enabling precise recognition and localization of infrared small targets.

Although RepViT was originally introduced as a lightweight backbone for general vision tasks, it exhibits particularly pronounced advantages in infrared small-target detection. Infrared targets typically contain extremely weak texture information and minimal structural details, making them highly susceptible to irreversible feature loss during the downsampling process in traditional convolutional networks. By leveraging the MetaFormer architecture, RepViT strengthens global contextual modeling while maintaining a lightweight design, enabling the network to better preserve faint thermal signatures across layers and thereby improving the discriminability of weak targets.

In addition, its structural re-parameterization mechanism allows the network to employ multi-branch structures during training to enhance the fusion of high-frequency and low-frequency information, while collapsing into a single-branch convolution during inference. This achieves a favorable balance between enhanced representational capability and real-time efficiency. Such characteristics align well with the “weak signal, strong background” nature of infrared imaging and effectively reduce the blurring and loss of small-target information during downsampling, ultimately improving detection stability and reliability.

### 3.3. C3k2_DyGhost Module

In YOLOv11, the C3k2 block is a built-in Cross Stage Partial (CSP) module used in both the backbone and neck. It follows the standard C3 framework, where the input feature map is split into two branches: one branch bypasses the block, and the other branch passes through a sequence of bottleneck layers. The suffix “k2” indicates that two 3 × 3 convolution-based bottleneck units are used inside the C3 block, which enhances feature extraction capacity while maintaining a lightweight structure. In this work, we adopt the original YOLOv11 C3k2 block as the baseline and replace its bottleneck part with the proposed DyGhostBottleneck to construct the C3k2_DyGhost module.

**Figure 4:**
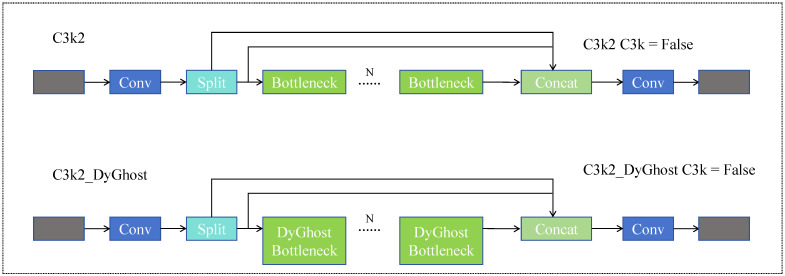
C3k2_DyGhost structure. The upper figure illustrates the architecture of the C3k2 module, while the lower figure presents the structure of the C3k2_DyGhost module. Both correspond to the case where C3k = False.

To further enhance the feature extraction capability of the model for infrared small-target detection tasks, this study designs an improved C3k2_DyGhost module based on the original C3k2 module in the YOLOv11n network. As shown in Figure 4, the module inherits the residual structure of the C3 framework to maintain the continuity of feature flow and the stability of gradient propagation. Here, the term “C3 framework” refers to the Cross Stage Partial C3 block used in the official YOLOv11 implementation. The core idea is to replace the original Bottleneck unit with a DyGhostBottleneck, which combines Ghost feature generation with dynamic convolution [39]. This structure enables adaptive feature modeling while preserving lightweight extraction efficiency. Similarly to the dynamic spectral–spatial perception mechanism explored in DSP-Net [40], the proposed DyGhost module leverages adaptive convolutional kernels to adjust feature extraction according to input distribution, thereby improving its resilience to complex infrared backgrounds.

The traditional Ghost Module generates intrinsic features using a small number of standard convolutions and then produces redundant features through linear transformations (such as depthwise or pointwise convolutions), thereby obtaining rich feature representations at a low computational cost. However, since its convolutional kernel weights are fixed, it lacks adaptability to variations in input feature distributions and struggles to effectively capture weak small targets in complex infrared backgrounds.

**Figure 5:**
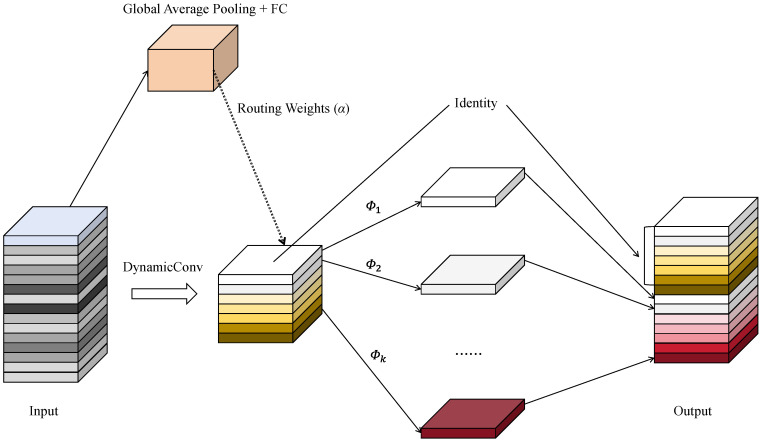
DyGhost Module structure.

To address this limitation, this study introduces a dynamic convolution mechanism into the Ghost feature generation stage, enabling input-adaptive feature modeling through multiple sets of learnable convolution kernels. The structure of the DyGhost module is shown in Figure 5. Specifically, the DyGhost module predefines *K* sets of convolutional kernel parameters and employs a lightweight attention branch to generate the corresponding weighting coefficients ${\left\{\alpha_{k}\right\}}_{k=1}^{k}$ (for (k = 1, 2, …, K)). These coefficients are produced by a Softmax function to ensure the balance and stability of kernel combinations.

Let the input feature be $X\in R^{C_{in}\times H\times W}$. The DyGhost module generates multiple feature responses using the *K* learnable convolution kernels ${\left\{W_{k}\right\}}_{k=1}^{K}$ as follows:

(4) $$Y_{k}=W_{k}*X,k=1,2,\ldots ,K$$

Here, $W_{k}$ denotes the *k*-th set of convolutional kernel parameters, $Y_{k}$ represents the corresponding feature response, and * indicates the convolution operation.

To achieve input-adaptive feature modeling, the DyGhost module introduces a lightweight routing branch, which first extracts global contextual information from the input via Global Average Pooling (GAP). Then, a linear mapping function $f_{k}$(·) is applied to generate the weighting coefficient $\alpha_{k}$:

(5) $$\alpha_{k}=\frac{\exp(f_{k}(GAP(X)))}{\sum_{j=1}^{K}\exp(f_{j}(GAP(X)))}$$

The final output feature is obtained by computing the weighted summation of the convolution results from all kernel groups:

(6) $$Y=\sum_{k=1}^{K}\alpha_{k}\cdot Y_{k}$$

This mechanism allows the model to adjust convolutional responses according to the input feature distribution, improving its ability to handle diverse targets and complex infrared backgrounds.

Subsequently, during the Ghost feature generation stage, the redundant features produced by the linear transformation are concatenated with the primary features:

(7) $$F_{ghost}=\left[Y,{DWConv}_{3\times 3}(Y)\right]$$

Here, $F_{ghost}$ denotes the concatenated Ghost feature map. The input features are then fused through batch normalization (*BN*) and residual connection:

(8) $$Z=BN(F_{ghost})+X$$

When the stride is set to 1, the residual branch directly connects the input and output to achieve the fusion of shallow and deep features. When the stride is 2, the input is first processed by a downsampling convolution to match the spatial dimensions.

**Figure 6:**
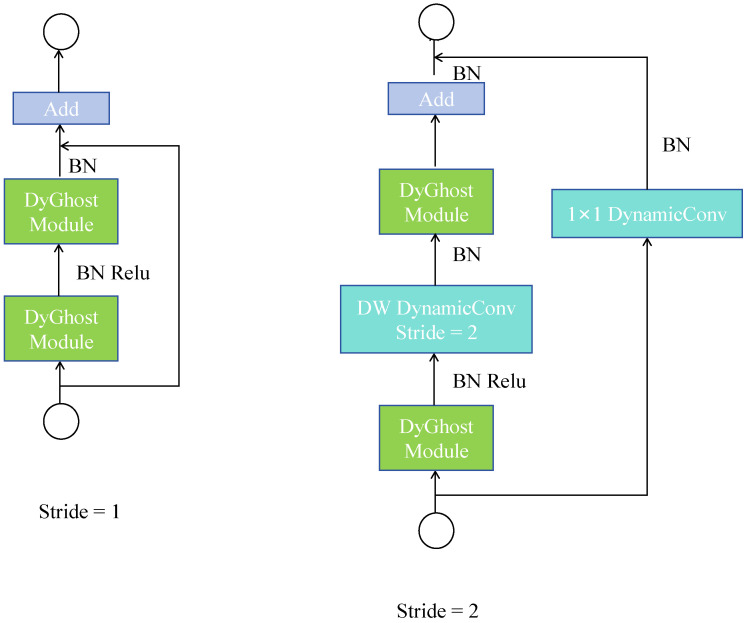
DyGhostBottleneck structure.

As illustrated in Figure 6, when the stride equals 1, the main branch consists of two stacked DyGhost modules, each following the standard Convolution Layer → Batch Normalization (*BN*) [41] → ReLU [42] activation structure to enable efficient feature extraction and nonlinear representation. The residual branch directly adds the input features—after *BN*—to the output of the main branch, thereby achieving effective fusion of shallow details and deep semantic information while maintaining computational efficiency.

In contrast, when the stride is 2, the main branch performs downsampling through a Depthwise Dynamic Convolution [43], resulting in the feature map resolution being reduced by half. Although this process helps enlarge the receptive field and capture higher-level semantic information, it inevitably leads to a significant loss of fine-grained textures and low-contrast thermal target details in UAV-based infrared small object detection tasks. In extreme cases, certain minute targets may even vanish completely from the feature maps. Therefore, this study adopts a stride = 1 configuration within the DyGhost module to maximally preserve spatial resolution and detailed features, ensuring stronger response capability and higher localization accuracy when detecting weak and small-scale infrared targets.

This structure maintains computational efficiency while enabling an effective integration of local details and global semantics during cross-layer feature propagation. By embedding dynamic convolution into the Ghost feature generation unit, the module enhances its capability to capture high-frequency texture and edge information while remaining lightweight. This property is particularly beneficial in infrared small target detection scenarios, which are often characterized by low contrast and high noise. The DyGhost module adaptively adjusts convolutional weights according to the saliency distribution of input features, thereby amplifying small target responses and suppressing background interference.

In summary, infrared scenes typically exhibit low contrast, strong background noise, and a large number of pseudo-targets, making the extraction of stable and discriminative small-target features particularly challenging. Although the Ghost module provides a lightweight computational framework, its fixed convolution kernels lack the ability to adapt to the complex and rapidly varying background characteristics of infrared imagery. As a result, the weak structural cues of small infrared targets are easily overwhelmed by dominant background textures or noise patterns, leading to missed detections and reduced feature reliability. By introducing dynamic convolution, the C3k2_DyGhost module enables the network to adaptively select the most suitable kernel combinations according to the input feature distribution. This mechanism helps retain weak target cues under low signal-to-noise conditions and reduces interference from non-target regions such as background heat sources and reflections.

With this adaptive feature modeling strategy, C3k2_DyGhost improves feature accuracy and stability compared with the original C3k2 structure, while keeping computational complexity and parameters at a low level. More importantly, its dynamic response characteristics closely align with the physical properties of infrared imaging, which relies on local energy fluctuations rather than the rich color or texture cues present in visible-light imagery. This makes C3k2_DyGhost particularly suitable for UAV-based infrared small-target detection, as it provides a robust, flexible, and highly discriminative feature extraction foundation for subsequent feature fusion and detection head prediction. The enhanced adaptability exhibited by this module offers valuable insights for the future design of lightweight feature extraction architectures tailored to infrared detection tasks.

### 3.4. Adaptive Fusion-IoU Loss

In object detection tasks, Bounding Box Regression (BBR) [44] is a critical component that directly determines detection accuracy, with the design of the loss function playing a central role. Traditional IoU-based losses (such as GIoU, DIoU, and CIoU) primarily describe the discrepancy between predicted and ground-truth boxes in terms of geometric metrics like center distance, aspect ratio, and bounding area. Although these methods alleviate issues such as gradient vanishing and scale insensitivity inherent in the original IoU formulation, improvements based purely on geometric constraints have gradually reached saturation. Moreover, the coupling among different geometric terms limits the optimization potential.

In UAV infrared small-target scenarios, high-quality prediction boxes are limited, and low-quality ones dominate the gradient updates. This imbalance slows convergence and reduces localization accuracy. To address this issue, this paper proposes an Adaptive Fusion-IoU Loss (AF-IoU), which redefines the weighting mechanism of predicted box quality. AF-IoU enables the model to dynamically adjust its focus between high- and low-quality prediction boxes at different training stages, thereby achieving a better balance between detection accuracy and training efficiency.

The core idea of AF-IoU goes beyond refining geometric metrics and establishes a “coarse-to-fine” training process through dynamic weight allocation. Its key mechanisms include a Scaled Focal Box strategy, annealing-based attention adjustment, and a confidence-weighted scheme [45]. Specifically, AF-IoU indirectly modifies the IoU value by scaling the predicted and ground-truth boxes, thereby adjusting the loss weights of predictions with varying quality. Let the original IoU be defined as:

(9) $${IoU}_{ori}=\frac{\left|B_{p}\cap B_{gt}\right|}{\left|B_{p}\cup B_{gt}\right|}$$

When the predicted box is shrunk, the intersection area decreases, IoU becomes smaller, and the loss increases; conversely, when the predicted box is expanded, IoU increases and the loss decreases. The scaling ratio *r* determines the focus of the model: when *r* < 1, the model emphasizes high-quality prediction boxes; when *r* > 1, it emphasizes low-quality ones. This approach achieves dynamic weight redistribution solely through scale variation, without introducing additional geometric computations, allowing the model to adaptively focus on more representative samples throughout different training stages.

To balance the rapid convergence in early training with precise regression in later stages, AF-IoU introduces a dynamic annealing strategy based on box attention. This strategy controls the scaling ratio through a dynamic hyperparameter ratio, which gradually decreases over training epochs, smoothly shifting the attention of the model from low-quality to high-quality prediction boxes. The evolution pattern follows a cosine annealing schedule [46], expressed as:

(10) $$r=0.75\times \cos\left(\frac{\pi \times epoch}{T}\right)+1.25$$

Here, *T* denotes the total number of training epochs. The dynamic scaling ratio *r* decreases from 2.0 to 0.5 over the total training epochs following a cosine-annealing schedule. A larger ratio at the early training stage enlarges the receptive range and guides the model to focus on low-quality prediction boxes, thereby accelerating convergence. The range of 2.0 → 0.5 was empirically determined through preliminary experiments to achieve a stable balance between convergence speed and localization accuracy. As training progresses and ratio gradually decreases, the model progressively shifts its attention toward high-quality prediction boxes, achieving more precise bounding box localization. This coarse-to-fine dynamic adjustment strategy effectively mitigates the issue of sample quality imbalance commonly encountered in object detection tasks.

In addition, AF-IoU incorporates the concept of Focal Loss by introducing a confidence-weighted term to further optimize the loss distribution. Let the confidence score of a predicted box be denoted as conf; the overall formulation of AF-IoU can be expressed as:

(11) $$L_{AF-IoU}=(1-IoU_{r}{)}^{\alpha }\cdot (1-conf{)}^{\gamma }$$

In Equation (11), ${IoU}_{r}$ denotes the IoU computed after isotropically scaling both the predicted box and the ground-truth box by the ratio r. The confidence term conf∈[0,1] acts as a soft weighting factor that increases the loss for low-confidence predictions.

(12) $${IoU}_{scaled}=\frac{\left|rB_{p}\cap rB_{gt}\right|}{\left|rB_{p}\cup rB_{gt}\right|}$$

where r represents the dynamic scaling factor. The parameter *γ* (set to 0.5 in this study) follows the focal-loss principle, which uses an exponential coefficient to emphasize hard samples. Following the concept proposed in Alpha-IoU [47], the parameter α (set to 1.5 in this study) acts as the IoU exponent that adjusts the weighting of high-quality predictions through a power-scaling mechanism. A larger α increases the gradient contribution of high-IoU samples and penalizes low-IoU predictions more strongly, thereby improving the precision of bounding-box regression. By jointly modeling IoU and confidence, AF-IoU achieves unified optimization of geometric scale, prediction confidence, and sample quality, leading to a more balanced weighting of high- and low-quality predictions throughout different training phases.

Infrared small targets typically occupy only a few pixels, making their bounding boxes extremely sensitive to even slight localization deviations. Under such conditions, traditional IoU-based loss functions often suffer from gradient domination by low-quality samples and insufficient optimization in the high-IoU region, which further amplifies localization errors for tiny targets. AF-IoU addresses this issue by introducing a dynamically weighted scaling mechanism that adaptively redistributes optimization focus according to the quality of prediction boxes. Its core design employs a cosine annealing schedule to smoothly adjust the scaling ratio throughout training, enabling the model to concentrate on low-quality predictions during the early stages to accelerate convergence toward coarse spatial structures. As training progresses, the weighting gradually shifts toward high-quality predictions, enhancing fine-grained regression accuracy for infrared small targets whose bounding boxes are highly sensitive to displacement.

This coarse-to-fine optimization strategy effectively mitigates gradient imbalance caused by sample quality disparity and ensures smoother, more stable gradient variation across the entire training process. Moreover, AF-IoU incorporates IoU exponentiation and confidence modulation, allowing the model to penalize boxes with larger positional deviations more strongly, thereby improving localization reliability in noisy infrared environments. Given that even a minimal regression error may render an infrared small target completely undetectable, the dynamic nature of AF-IoU makes it inherently more suitable for infrared small-target localization than conventional IoU-based losses. At the same time, AF-IoU maintains full compatibility with standard IoU loss formulations and can be directly substituted for CIoU in the YOLO series without any architectural modification, offering both refined optimization capability and strong generalizability for high-quality bounding-box regression tasks.

## 4. Experiments

### 4.1. Datasets

#### 4.1.1. HIT-UAV

We trained and evaluated IFD-YOLO using the publicly available HIT-UAV [48] dataset. The HIT-UAV dataset, developed by the research team at Harbin Institute of Technology (HIT), aims to provide a reliable data foundation for object detection and tracking under nighttime conditions. Its key characteristic lies in focusing on nighttime surveillance detection and tracking, leveraging infrared thermal imaging to overcome the limitations of conventional visible-light cameras in low-illumination environments.

**Figure 7:**
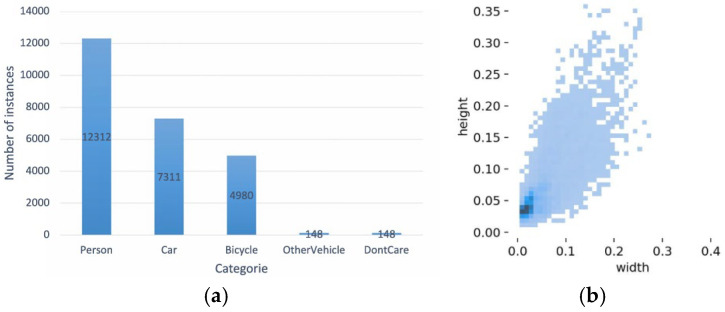
Visualization of the HIT-UAV dataset used in this study. (**a**) The distribution of object categories and the number of instances per category. (**b**) The distribution of bounding box aspect ratios in the original images, where each point represents a labeled object with its corresponding width and height proportions.

The dataset comprises 2898 infrared thermal images extracted from 43,470 UAV video frames, covering diverse scenes such as campuses, parking lots, roads, and playgrounds under varying illumination conditions from daytime to nighttime. The dataset is divided into training sets, validation sets, and test sets in a ratio of 7:2:1, respectively. HIT-UAV contains five object categories with a total of 24,899 annotated instances. As illustrated in Figure 7a, person, car, and bicycle constitute the majority of the targets. Figure 7b depicts the size distribution of targets in the images, where most are small and occupy only a few dozen pixels. The absence of color and rich texture features, combined with the prevalence of small targets, provides a challenging benchmark for evaluating the capability of the algorithm in infrared small target detection.

#### 4.1.2. IRSTD-1k

Furthermore, the IRSTD-1K [49] dataset was employed for additional performance evaluation of IFD-YOLO. This dataset consists of 1001 real-world infrared images captured by infrared cameras, with each manually annotated at the pixel level. All images have a fixed resolution of 512 × 512. IRSTD-1K includes a wide range of small targets—such as UAVs, biological entities, ships, and vehicles—captured under varying distances and positions. The dataset is divided into a training set (640 images), a validation set (160 images), and a test set (201 images). As a single-class dataset containing approximately 10,000 annotated targets, IRSTD-1K encompasses diverse backgrounds, including oceans, rivers, fields, mountains, urban areas, and cloudy conditions. These scenes exhibit significant clutter and noise, making IRSTD-1K a comprehensive benchmark for evaluating infrared small target detection algorithms.

### 4.2. Implementation Details

All experiments are implemented using the PyTorch 2.6.0 framework, with YOLOv11n serving as the baseline model. The experiments are conducted on a system equipped with an Intel i5-12600KF CPU, Windows 11 OS, and an NVIDIA RTX 4070 SUPER GPU (12 GB VRAM), with CUDA version 12.6 and PyCharm 2024.3.3 as the development environment. To ensure a fair comparison, the proposed IFD-YOLO and all compared methods (RT-DETR, Picodet, YOLOv8n, YOLO-MBL, YOLO-MARS, YOLO-SRMX, etc.) are trained from scratch without any external pretrained weights. Unless otherwise specified, all models are re-trained under a unified configuration: the input image size is fixed at 512 × 512, the initial learning rate is set to 0.01, the batch size is 16, and the number of training epochs is 200. Model optimization is performed using the Stochastic Gradient Descent (SGD) algorithm.

The detailed hardware configuration and training parameters are summarized in Table 1 and Table 2, respectively.

### 4.3. Experimental Evaluation Metrics

To evaluate the detection performance of the proposed model, this study adopts R*ecall* (R), *Precision* (P), mean Average Precision (*mAP*), number of parameters, and model size as the evaluation metrics for UAV-based aerial image detection. Specifically, mAP@50 represents the mean of the average precision (*AP*) values across all classes when the IoU threshold is 0.5, while mAP@50:95 denotes the average of *mAP* values computed at IoU thresholds ranging from 0.5 to 0.95 with a step size of 0.05. The inference speed, measured in frames per second (FPS), is also reported to evaluate the real-time performance of the model. The corresponding calculation formulas are shown in Equations (13)–(16).

(13) $$Recall=\frac{TP}{TP+FN}$$

(14) $$Precision=\frac{TP}{TP+FP}$$

(15) $$AP=\int_{0}^{1}P(R)dR$$

(16) $$mAP=\frac{1}{n}\sum_{i=1}^{n}AP_{i}$$

Here, *TP* (True Positives) denotes the number of correctly predicted positive samples, *FP* (False Positives) denotes the number of negative samples incorrectly predicted as positive, and *FN* (False Negatives) represents the number of positive samples incorrectly predicted as negative. *P*(*R*) denotes the *Precision*–*Recall* function.

### 4.4. Experimental Results

#### 4.4.1. Comparative Analysis of Different Network Backbone

As shown in Table 3, multiple comparative experiments were conducted to investigate the impact of different backbone networks on the performance of the proposed IFD-YOLO framework. The backbone serves as the core feature extraction component of an object detection model, and its design directly determines the feature representation capability and overall computational complexity of the model. To ensure fair comparison, all experiments maintained identical detection heads, neck structures, and training parameters, with only the backbone network being replaced while keeping all other factors unchanged. Using the baseline model (denoted by *) as a reference, its evaluation metrics include a precision of 85.9%, recall of 77.7%, mAP@50 of 83.4%, mAP@50:95 of 54.2%, 2.8 M parameters, and a computational complexity of 6.7 GFLOPs.

Specifically, this study compares several mainstream backbone architectures, including StarNet, TransNeXt, VanillaNet, EfficientNetV2, LSKNet, and RevColV1, against the adopted RepViT network to evaluate their impact on model performance. The experimental results reveal significant differences among these backbones in terms of detection accuracy, parameter size, and computational cost.

Among them, RepViT achieves the best balance between performance and efficiency. It attains a precision of 88.6%, recall of 78.4%, mAP@50 of 85.9%, and mAP@50:95 of 55.2%, while maintaining an extremely lightweight configuration with 1.97 M parameters and 5.0 GFLOPs. This demonstrates that RepViT effectively integrates the local receptive capability of convolutional networks with the global modeling capacity of Transformers, enabling it to capture more discriminative infrared small-target features while remaining computationally efficient.

StarNet has the smallest parameter count at 1.87 M and low computational load of 5.7 GFLOPs, yet its accuracy drops markedly, with mAP@50 falling to 79.3% and mAP@50:95 decreasing to 50.2%. TransNeXt achieves a slightly higher recall of 77.8%, but the improvement has little impact on overall precision. VanillaNet offers a moderate balance between accuracy and computational cost, reaching an mAP@50 of 84.4% and an mAP@50:95 of 54.8%, although its complexity increases noticeably to 15.6 GFLOPs. EfficientNetV2 obtains the highest recall at 80.7%, but its relatively low precision of 72.1% weakens its overall *mAP* performance. LSKNet performs similarly to the baseline, accompanied by a moderate rise in computational cost to 7.9 GFLOPs. RevColV1 achieves the highest accuracy among all compared backbones, reaching an mAP@50 of 86.5% and an mAP@50:95 of 56.8%; however, the model demands extremely high computational resources, with parameters increasing to 32.8 M and FLOPs to 78.9 GFLOPs, making it impractical for UAV deployment.

In summary, the results validate that RepViT significantly enhances the feature representation capability of the model while preserving a lightweight design, achieving an excellent trade-off between detection accuracy and computational efficiency. Consequently, RepViT is adopted as the final backbone network for the IFD-YOLO architecture.

#### 4.4.2. Comparative Analysis of Different Loss Functions

Table 4 compares several IoU-based bounding-box regression loss functions within the improved IFD-YOLO framework to examine their influence on infrared small-target detection. All experiments were conducted under identical conditions, keeping the backbone, neck, and training configurations unchanged. The baseline model, which incorporates all improvements except the proposed AF-IoU, achieved a precision of 87.5%, a recall of 73.9%, an mAP@50 of 84.6%, and an mAP@50:95 of 54.6%, serving as the reference for subsequent comparisons.

Compared with the baseline, GIoU and EIoU produced slightly lower performance. GIoU reduced precision by 7.9% and mAP@50 by 1.8%, indicating that its penalty formulation is less effective for compact and low-contrast infrared targets. EIoU improved recall to 79.4% but provided almost no gain in mAP@50:95, suggesting limited optimization capability for high-IoU predictions. Wise-IoU achieved the highest recall of 82.6%, outperforming the baseline by 8.7%, but its precision dropped sharply to 76.7%, resulting in only marginal improvement in overall accuracy. MPDIoU maintained performance similar to the baseline, with a precision of 87.8% and mAP@50 of 82.4%. Although its results were stable, the absence of improvement in mAP@50:95 indicates limited benefit for high-quality bounding boxes. Shape-IoU provided a modest increase in both precision and recall, reaching 88.2% and 78.9%, respectively, with an mAP@50 of 84.5%, nearly matching the baseline while improving the detection balance. Focal-IoU emphasized difficult samples but failed to generalize effectively, recording the lowest precision of 71.4% despite a relatively high recall of 81.1%, resulting in the weakest overall *mAP*.

When AF-IoU is introduced, all four configurations outperform the baseline in overall detection accuracy. When the scaling factor r decreases from 1.5 to 0.75, the detector obtains a precision of 88.3% and an mAP@50 of 85.4%. This setting slightly improves the baseline and keeps a good balance between precision and recall. When r decreases from 2.0 to 0.5, the detector achieves the best results: precision and recall reach 88.6% and 78.4%, and mAP@50 and mAP@50:95 reach 85.9% and 55.2%. This indicates that a moderate coarse-to-fine scaling schedule is most effective for emphasizing high-quality predictions.

Increasing the upper bound of r to make it decrease from 2.5 to 0.5 further raises recall to 79.1%, the highest among all configurations, but slightly reduces precision to 87.2%. This suggests that an overly large initial scaling introduces more noisy low-quality samples. In contrast, restricting r to a high range and only decreasing it from 2.0 to 1.0 yields the weakest improvement, with mAP@50 and mAP@50:95 reduced to 84.2% and 54.1%. This result shows that insufficient emphasis on high-IoU samples limits the benefit of AF-IoU and confirms the necessity of a full coarse-to-fine optimization process.

In summary, the experimental results reveal clear performance differences among IoU-based loss functions for infrared small-target detection. Most existing losses struggle to simultaneously optimize high-quality bounding boxes and maintain a balanced precision–recall trade-off. In contrast, AF-IoU consistently surpasses the baseline under all scaling configurations, with the configuration where the scaling factor (*r*) is gradually reduced from 2.0 to 0.5 during training achieving the best overall results. These findings demonstrate that dynamically adjusting the contribution of high- and low-quality predictions through a coarse-to-fine scaling strategy is an effective way to enhance both accuracy and robustness. This also confirms the necessity and effectiveness of AF-IoU as the core regression loss within the IFD-YOLO framework.

#### 4.4.3. Comparison with Baseline Models

To assess the effectiveness and applicability of the improved model for infrared small target detection in UAV scenarios, comparative experiments were conducted under identical conditions using the baseline model. Both experiments were trained from scratch without any pre-training. The results demonstrate that, compared with the original model, the improved version achieves notable performance gains on HIT-UAV. Specifically, *Precision* and *Recall* both increased to varying degrees, mAP@50 and mAP@50:95 improved by 4.9% and 3.1%, respectively, while the number of parameters and computational cost were reduced by 23% and 20%, respectively. These results indicate that the proposed model not only exhibits higher detection accuracy but also achieves lower computational complexity and a smaller model size, making it particularly well-suited for resource-constrained UAV platforms. Overall, the experimental results validate the effectiveness of the proposed approach. The quantitative results are summarized in Table 5.

**Figure 8:**
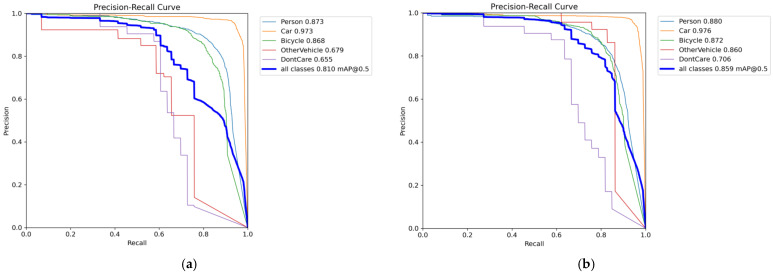
*Precision*–*Recall* (PR) curves of different models on HIT-UAV. (**a**) YOLOv11n. (**b**) IFD-YOLO. Each colored line represents the PR curve of one object category, and the bold blue line indicates the overall PR curve across all classes.

Figure 8 present the comparison of *Precision*–*Recall* (PR) curves between the baseline YOLOv11 model and the proposed IFD-YOLO model on HIT-UAV. Overall, IFD-YOLO demonstrates superior detection performance across all categories compared to the baseline model. The overall mAP@0.5 increased from 0.810 to 0.859, indicating a clear improvement in detection precision. In particular, IFD-YOLO achieves substantial performance gains in challenging categories such as OtherVehicle and DontCare, while also yielding moderate improvements in other categories. The PR curves shift consistently toward the upper-right corner, reflecting higher precision and recall. These findings confirm that the proposed architectural enhancements significantly strengthen the ability of the model to extract and discriminate infrared small-target features, leading to improved detection accuracy and adaptability.

Furthermore, to validate the generalization ability and applicability of the proposed IFD-YOLO model in infrared small target detection, additional comparative experiments were performed on the IRSTD-1K dataset against the baseline YOLOv11n model. The results, presented in Table 6, show that the improved model outperforms the baseline across all metrics: *Precision* (P) increased from 81.8% to 85.4%, *Recall* (R) from 60.7% to 66.2%, mAP@50 from 73.3% to 78.3%, and mAP@50:95 from 34.3% to 36.4%. These results verify that the proposed improvements not only enhance detection performance and maintain lightweight efficiency but also exhibit strong generalization capability and practical applicability in infrared small target detection tasks.

**Figure 9:**
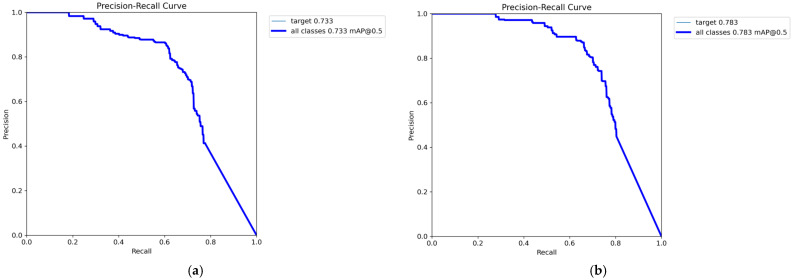
*Precision*–*Recall* (PR) curves of different models on IRSTD-1k. (**a**) YOLOv11n. (**b**) IFD-YOLO. The blue lines represent the PR curves for the single target category in IRSTD-1k, with the bold curve indicating the overall PR performance.

Figure 9 illustrate the PR curve comparisons between the baseline and improved models on IRSTD-1K. Since this dataset contains a single target category, it provides a more direct assessment of infrared small target detection performance. The baseline model achieved an overall mAP@0.5 of 73.3%, while the improved model reached 78.3%, demonstrating a clear improvement in detection accuracy. More specifically, the improved model achieved a significant increase in *Recall* while maintaining a high level of *Precision*, resulting in a PR curve that shifts noticeably toward the upper-right region. This indicates that the model can capture more targets while reducing false detections. Overall, the proposed structural improvements substantially enhance the feature extraction and representation capabilities of the model, enabling higher accuracy in complex backgrounds and small-target detection scenarios while maintaining lightweight efficiency.

#### 4.4.4. Comparison with State-of-the-Art Methods

To comprehensively verify the superiority and effectiveness of the proposed IFD-YOLO model, comparative experiments were conducted against several state-of-the-art (SOTA) models and representative infrared small target detection methods on HIT-UAV. The results are summarized in Table 7.

As shown in Table 7, the proposed IFD-YOLO model exhibits significant performance advantages across all evaluation metrics. Specifically, IFD-YOLO achieves an mAP@50 of 85.9%, surpassing mainstream models such as YOLOv11n, YOLOv8n, and RT-DETR by 4.9%, 9.0%, and 7.3%, respectively. Meanwhile, it attains an mAP@50:95 of 55.2%, demonstrating stronger representation capability in precise localization and boundary regression of small targets. Moreover, the model maintains excellent efficiency, with only 1.97 M parameters and 5.0 GFLOPs, while achieving the highest inference speed of 210 FPS, indicating superior lightweight characteristics and real-time detection performance.

In addition, when compared with current SOTA infrared detection models such as PHSI-RTDETR, YOLO-MBL, YOLO-MARS and YOLO-SRMX, IFD-YOLO achieves the highest average detection accuracy and the best real-time capability while maintaining the lower parameter count and computational cost.

As shown in Table 8, the proposed IFD-YOLO achieves outstanding detection performance on the IRSTD-1k dataset. Specifically, it reaches an mAP@50 of 78.3% and an mAP@50:95 of 36.4%, outperforming lightweight detectors such as PicoDet, YOLOv8n, and YOLOv11n by large margins in both accuracy and precision. IFD-YOLO achieves high accuracy while maintaining exceptional efficiency, requiring only 1.97 M parameters and 5.0 GFLOPs and delivering an inference speed of approximately 210 FPS. In comparison, heavier detectors such as RT-DETR demand 19.97 M parameters and 100.9 GFLOPs, highlighting the clear advantage of the proposed model.

In addition, when compared with existing state-of-the-art infrared detection networks such as PHSI-RTDETR, YOLO-MBL, YOLO-MARS and YOLO-SRMX, IFD-YOLO achieves comparable or higher accuracy while preserving the smallest model size and computational cost, demonstrating its excellent trade-off between detection precision and efficiency for UAV-based infrared small-target scenarios.

In summary, IFD-YOLO achieves a remarkable balance between detection accuracy and computational efficiency, demonstrating strong practicality and engineering applicability.

#### 4.4.5. Ablation Studies and Analysis

To evaluate the contribution of each proposed module to the overall performance on HIT-UAV, seven groups of ablation experiments were conducted. The baseline model was YOLOv11n, and all experiments were performed under identical settings. The RepViT backbone, C3k2_DyGhost module, and AF-IoU loss function were gradually introduced for comparison, as indicated by the checkmarks (√) in Table 9.

From the ablation results in Table 9, each proposed component contributes positively to the overall detection performance of infrared small targets in UAV imagery. The baseline YOLOv11n model achieves mAP@50 and mAP@50:95 scores of 81.0% and 52.1%, respectively. After incorporating the RepViT backbone, the mAP@50 increases to 83.3%. This improvement suggests that RepViT strengthens the joint modeling of local details and global semantics while preserving a lightweight architecture. Replacing the original C3k2 module with the C3k2_DyGhost module further improves mAP@50 to 82.6%, demonstrating that the dynamic convolution and lightweight design strengthen fine-grained feature extraction and cross-layer information fusion. These enhancements are particularly beneficial for small infrared target detection.

Furthermore, substituting the original bounding box regression loss with the proposed Adaptive Fusion IoU (AF-IoU) loss improves mAP@50 and mAP@50:95 to 84.2% and 54.5%, respectively, confirming its effectiveness in enhancing localization and matching precision.

Notably, when all three strategies are integrated, the resulting IFD-YOLO achieves 85.9% mAP@50 and 55.2% mAP@50:95, outperforming the baseline by 4.9% and 3.1%, respectively, while keeping parameters and GFLOPs as low as 2.0 M and 5.0 G.

These improvements highlight the complementary and synergistic effects of the three modules: RepViT enhances representation capacity, C3k2_DyGhost improves feature preservation and fusion, and AF-IoU refines bounding box regression. Together, they achieve an optimal balance among detection accuracy, model compactness, and convergence stability, validating the rationality and effectiveness of the proposed improvements.

### 4.5. Analysis of Detection Performance

**Figure 10:**
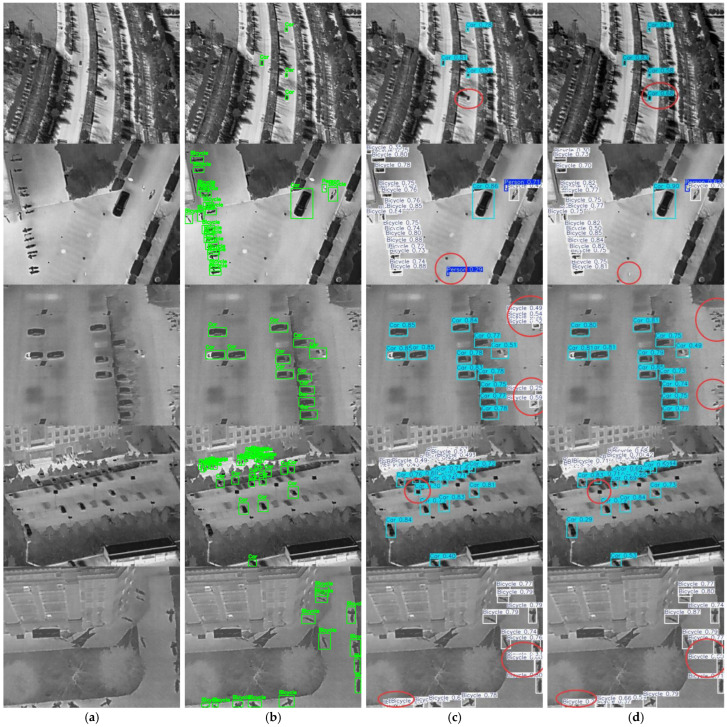
Comparison of detection results on the HIT-UAV dataset. (**a**) Original images; (**b**) Image with bounding boxes; (**c**) YOLOv11n; (**d**) IFD-YOLO. Note: The red circles highlight the regions of interest. The visualization results are presented only on the HIT-UAV dataset because IRSTD-1k does not contain UAV-based infrared scenes. As the IRSTD-1k dataset mainly consists of fixed-ground imaging conditions rather than aerial perspectives, it is less suitable for demonstrating the UAV-oriented detection capability of the proposed model.

To intuitively verify the detection performance of the improved model on the HIT-UAV dataset, five groups of infrared aerial images captured from different viewpoints and backgrounds were selected for comparison between the baseline YOLOv11n model and the proposed IFD-YOLO model. As shown in Figure 10, the baseline model exhibited varying degrees of false detections and missed detections in the first four groups of images. Specifically, the first and second groups showed missed and false detections, respectively. In the third group, a large area of false detections appeared on the right side of the output image processed by the baseline model. In the fourth group, the YOLOv11n model incorrectly identified streetlights in the parking lot as vehicles. Finally, in the fifth group, numerous overlapping bounding boxes were observed in the detection results of the baseline model.

In contrast, the proposed IFD-YOLO model demonstrates significantly better performance on the same samples. Specifically, the images processed by IFD-YOLO show no missed detections, primarily due to the introduction of the RepViT backbone. Through the convolutional re-parameterization mechanism, this backbone enhances the representational capability of the network while maintaining a lightweight structure. Consequently, the model can extract richer and more stable target features from weak-textured infrared backgrounds, thereby substantially improving its perception ability for small targets.

For the second, third, and fourth groups of images, the false detection problem was greatly alleviated with the introduction of the C3k2_DyGhost module. This module integrates adaptive dynamic convolution and ghost feature generation mechanisms to achieve efficient feature representation and suppress redundant information. By dynamically adjusting convolutional kernel weights according to the contextual information of the input image, the model enhances its discrimination between real targets and complex background regions, effectively reducing the false detection rate.

Finally, in the fifth group of images, the IFD-YOLO model eliminated the overlapping bounding boxes observed in the baseline model, benefiting from the adoption of the AF-IoU loss function. This loss introduces a unified IoU-constrained optimization strategy that jointly considers regression error, scale mismatch, and angular deviation, enabling more balanced and stable bounding box regression. As a result, the accuracy and consistency of the predicted bounding boxes are substantially improved.

In summary, the improved IFD-YOLO model reduces missed detections, suppresses false alarms, and mitigates bounding-box overlaps, demonstrating its effectiveness for infrared small-target detection in UAV-based aerial scenarios.

## 5. Discussion

The experimental results on the HIT-UAV and IRSTD-1k datasets show that the proposed IFD-YOLO framework improves infrared small-target detection. It also achieves a more balanced trade-off among accuracy, efficiency, and model complexity compared with lightweight infrared-specific detectors such as PHSI-RTDETR, YOLO-MBL, YOLO-MARS, and YOLO-SRMX.

On the IRSTD-1k dataset, YOLO-MBL achieves an mAP@50 of 78.4% with 2.40 M parameters and 5.7 GFLOPs. YOLO-MARS reports 77.5% mAP@50 with 3.32 M parameters and 8.7 GFLOPs, whereas YOLO-SRMX uses 11.42 M parameters and 10.0 GFLOPs but achieves only 75.6% mAP@50. The transformer-based PHSI-RTDETR attains 76.2% mAP@50 with 13.87 M parameters, 47.5 GFLOPs, and an inference speed of 114 FPS.

In contrast, IFD-YOLO achieves a competitive mAP@50 of 78.3% using only 1.97 M parameters and 5.0 GFLOPs and reaches the highest inference speed of 210 FPS; a similar pattern appears on the HIT-UAV dataset, where IFD-YOLO with the same lightweight configuration obtains 85.9% mAP@50 and outperforms PHSI-RTDETR, which achieves 83.4% mAP@50 with 13.87 M parameters, 47.5 GFLOPs, and an inference speed of 114 FPS. These comparisons show that IFD-YOLO achieves accuracy comparable to or higher than existing infrared-oriented detectors, especially when compared with the heavier PHSI-RTDETR. At the same time, it uses fewer parameters, requires less computation, and offers higher inference speed.

A similar advantage is observed on HIT-UAV, where IFD-YOLO reaches 85.9% mAP@50 and 55.2% mAP@50:95, outperforming YOLOv11n by a clear margin while preserving a lightweight design. These consistent results across datasets confirm that the integration of the RepViT backbone, the C3k2_DyGhost module, and the AF-IoU loss function provides complementary benefits for capturing weak infrared target cues, enhancing discriminative feature representation, and refining high-IoU bounding-box regression, ultimately enabling the model to deliver state-of-the-art performance while remaining suitable for UAV deployment.

A closer examination of the model design offers scientific insight into why these improvements occur. RepViT increases the ability of the network to capture long-range semantic relationships without sacrificing lightweight efficiency, which is crucial for handling low-contrast and blurred infrared targets. The C3k2_DyGhost module introduces dynamic convolutional adaptation that strengthens feature activation in cluttered or noisy backgrounds, effectively enhancing the robustness of model in complex infrared environments. Furthermore, AF-IoU optimizes the regression process through adaptive weighting, enabling more stable convergence toward precise localization. The performance gains observed in the ablation experiments, especially in high-IoU metrics, validate the effectiveness of these mechanisms and align with previous studies emphasizing the importance of attention modeling and IoU-based optimization in infrared small-target tasks.

Although IFD-YOLO demonstrates strong performance, several limitations should be acknowledged to provide a comprehensive understanding of its practical applicability. The model, while lightweight compared with existing infrared-specific detectors, still lacks embedded deployment verification on UAV onboard processors. Its real-time capability on GPU platforms does not necessarily guarantee similar performance in low-power embedded environments, and future work should include tests on representative edge hardware to assess inference speed, memory usage, and energy consumption. Another challenge lies in scenarios involving extreme noise, heavy occlusion, or rapid target movement. While the proposed modules alleviate some of these issues, the model may still struggle in highly degraded or dynamic infrared environments. More advanced multi-scale attention modeling or temporal information integration may be necessary to further strengthen robustness.

In summary, IFD-YOLO demonstrates clear advantages in accuracy, computational efficiency, and lightweight design, making it a strong candidate for UAV-based infrared small-target detection. However, improving embedded deployability, enhancing robustness under extreme scenarios, and incorporating more diverse infrared datasets remain important directions for future research. Further exploration of model compression, hardware–software co-design strategies, and expanded multi-scene UAV datasets will be essential to fully unlock the potential of IFD-YOLO in real-world aerial infrared applications.

## 6. Conclusions

This paper proposes IFD-YOLO, an improved infrared small-target detection algorithm based on YOLOv11n, addressing the challenges of small target size, complex backgrounds, low signal-to-noise ratio, and limited computational resources in UAV infrared imagery. The proposed framework introduces systematic enhancements from three aspects: A lightweight RepViT backbone that strengthens the joint modeling of local details and global semantics; A C3k2_DyGhost module that employs dynamic convolution and Ghost feature generation for adaptive feature extraction and fusion, enhancing the perception of weak infrared targets; An Adaptive Fusion IoU (AF-IoU) loss that dynamically balances sample weights during training, improving high-quality box regression accuracy and convergence stability.

The experimental results demonstrate that IFD-YOLO achieves superior performance on both the HIT-UAV and IRSTD-1K datasets, surpassing the baseline YOLOv11n by 4.9% in mAP@50 and 3.1% in mAP@50:95, while reducing the number of parameters and the computational cost by 23% and 21%, respectively. The model maintains excellent lightweight characteristics and inference efficiency, effectively reducing missed and false detections under complex infrared conditions, thereby demonstrating its robustness and superiority in infrared small-target detection.

In future work, we will deploy IFD-YOLO on embedded UAV platforms to assess its real-time performance under onboard computational constraints and explore software optimization combined with hardware acceleration to improve edge inference efficiency. We also plan to enhance the robustness of model in scenarios involving extreme noise, severe occlusion, or rapid target motion by investigating advanced multi-scale attention mechanisms and temporal information modeling. In addition, expanding infrared datasets and developing multi-scene UAV benchmarks will help improve generalization. Finally, model compression and hardware–software co-design strategies will be explored to further support efficient deployment of IFD-YOLO in real-world aerial infrared applications.

## Figures and Tables

**Table 1:** Experiential environment.

Component	Specification
Operating System	Windows11
CPU	Intel^®^Core™i5-12600KF
GPU	RTX 4070SUPER 12g
Programming Environment	Python 3.12
CUDA	12.6
Deep Learning Framework	Pytorch 2.6.0
IDE/Compiler	Pycharm 2024.3.3

**Table 2:** Training parameters.

Parameter	Value
batch size	16
Initial learning rate(lr0)	0.01
Final learning rate (lr1)	0.01
workers	8
optimizer	SGD
momentum	0.937

**Table 3:** Comparative Experiments with Different Network Backbone on HIT-UAV.

Backbone	P/%	R/%	mAP@50/%	mAP@50:95/%	Params/M	GFLOPs/G
*	85.9	77.7	83.4	54.2	2.80	6.7
* + StarNet [50]	84.2	71.7	79.3	50.2	**1.87**	5.7
* + TransNeXt [51]	78.5	77.8	79.6	53.8	3.10	7.8
* + VanillaNet [52]	83.3	76.5	84.4	54.8	4.70	15.6
* + EfficientNetV2 [53]	72.1	**80.7**	80.2	51.2	2.70	6.2
* + LSKNet [54]	81.2	74.6	82.5	53.4	3.20	7.9
* + RevColV1 [55]	84.6	76.2	**86.5**	**56.8**	32.8	78.9
* + RepViT	**88.6**	78.4	85.9	55.2	1.97	**5.0**

Note: ‘*’ represents YOLOv11n applying all improvements in this paper except the RepViT Backbone. Bold values indicate the best results in each respective column.

**Table 4:** Comparative Experiments with Different Loss Functions on HIT-UAV.

Loss Function	P/%	R/%	mAP@50/%	mAP@50:95/%
*	87.5	73.9	84.6	54.6
* + GIoU	79.6	74.1	82.8	53.2
* + EIoU	82.2	79.4	83.4	54.6
* + Wise-IoU [56]	76.7	**82.6**	83.8	53.4
* + MPDIoU [57]	87.8	76.3	82.4	54.2
* + Shape-IoU [58]	88.2	78.9	84.5	54.8
* + Focal-IoU [59]	71.4	81.1	81.4	52.2
* + AF-IoU(r:1.5 → 0.75)	88.3	77.9	85.4	54.7
* + AF-IoU(r:2.0 → 0.5)	**88.6**	78.4	**85.9**	**55.2**
* + AF-IoU(r:2.5 → 0.5)	87.2	79.1	85.6	54.9
* + AF-IoU(r:2.0 → 1.0)	86.3	76.1	84.2	54.1

Note: ‘*’ represents YOLOv11n applying all improvements in this paper except AF-IoU; “r:a → b” in parentheses indicates that the scaling factor r is gradually varied from a to b during training. Bold values indicate the best results in each respective column.

**Table 5:** Algorithm comparison experiment with baseline on HIT-UAV.

Network	P/%	R/%	mAP@50/%	mAP@50:95/%	Params/M	GFLOPs/G
YOLOv11n	83.9	73.7	81.0	52.1	2.58	6.3
IFD-YOLO	**88.6**	**78.4**	**85.9**	**55.2**	**1.97**	**5.0**

Note: Bold values indicate the best results in each respective column.

**Table 6:** Algorithm comparison experiment with baseline on IRSTD-1k.

Network	P/%	R/%	mAP@50/%	mAP@50:95/%	Params/M	GFLOPs/G
YOLOv11n	81.8	60.7	73.3	34.3	2.58	6.3
IFD-YOLO	**85.4**	**66.2**	**78.3**	**36.4**	**1.97**	**5.0**

Note: Bold values indicate the best results in each respective column.

**Table 7:** Algorithm comparison experiment on HIT-UAV.

Network	mAP@50/%	mAP@50:95/%	Params/M	GFLOPs/G	FPS/Hz
RT-DETR	78.6	48.7	19.97	100.9	42
Picodet	70.6	38.9	**0.99**	**1.08**	-
YOLOv8n	76.9	46.3	3.12	8.3	187
YOLOv11n	81.0	52.1	2.58	6.3	192
PHSI-RTDETR [26]	83.4	53.6	13.87	47.5	114
YOLO-MBL [60]	84.1	54.8	2.40	5.7	184
YOLO-MARS [61]	83.2	53.8	3.32	8.7	175
YOLO-SRMX [62]	82.8	53.1	11.42	10.0	130
IFD-YOLO	**85.9**	**55.2**	1.97	5.0	**210**

Note: The FPS of PicoDet is not reported, as it is primarily optimized for mobile and edge devices rather than GPU-based inference. All FPS values are measured on the RTX 4070 Super GPU with an input resolution of 512 × 512. Bold values indicate the best results in each respective column.

**Table 8:** Algorithm comparison experiment on IRSTD-1k.

Network	mAP@50/%	mAP@50:95/%	Params/M	GFLOPs/G	FPS/Hz
RT-DETR	71.6	30.2	19.97	100.9	42
Picodet	67.4	29.3	**0.99**	**1.08**	-
YOLOv8n	74.9	35.4	3.12	8.3	187
YOLOv11n	73.3	34.3	2.58	6.3	192
PHSI-RTDETR [26]	76.2	35.7	13.87	47.5	114
YOLO-MBL [60]	78.4	37.0	2.40	5.7	184
YOLO-MARS [61]	77.5	**36.6**	3.32	8.7	175
YOLO-SRMX [62]	75.6	35.9	11.42	10.0	130
IFD-YOLO	**78.3**	36.4	1.97	5.0	**210**

Note: The FPS of PicoDet is not reported, as it is primarily optimized for mobile and edge devices rather than GPU-based inference. All FPS values are measured on the RTX 4070 Super GPU with an input resolution of 512 × 512. Bold values indicate the best results in each respective column.

**Table 9:** Ablation comparison experiment.

YOLOv11n	RepViT	C3k2_DyGhost	AF-IoU	P/%	R/%	mAP@50/%	mAP@50:95/%	Params/M	GFLOPs/G
√				83.9	73.7	81.0	52.1	2.6	6.3
√	√			85.8	76.0	83.3	53.4	**1** **.7**	**4** **.1**
√		√		84.7	74.0	82.6	53.9	2.8	6.7
√			√	84.6	76.9	82.4	53.6	2.6	6.3
√	√	√		87.5	73.9	84.6	54.6	2.0	5.0
√	√		√	86.2	75.5	84.2	54.5	**1** **.7**	**4** **.1**
√	√	√	√	**88** **.6**	**78.4**	**85.9**	**55.2**	2.0	5.0

Note: √ indicates that the corresponding module is included in the ablation setting. Bold values indicate the best results in each respective column.

## Data Availability

The original HIT-UAV dataset and the IRSTD-1k dataset, which were analyzed in this study, are publicly available at https://doi.org/10.1038/s41597-023-02066-6 and https://doi.org/10.1109/CVPR52688.2022.00095. The specific refined subsets of these datasets (including their partitioning into training, validation, and test sets), along with the source code for the IFD-YOLO model and its trained weights, are available from the corresponding author upon reasonable request.
